# Effects of Early Life Stress on the Developing Basolateral Amygdala-Prefrontal Cortex Circuit: The Emerging Role of Local Inhibition and Perineuronal Nets

**DOI:** 10.3389/fnhum.2021.669120

**Published:** 2021-08-26

**Authors:** Angela Guadagno, Claudia Belliveau, Naguib Mechawar, Claire-Dominique Walker

**Affiliations:** ^1^Douglas Mental Health University Institute, Montreal, QC, Canada; ^2^Department of Psychiatry, McGill University, Montreal, QC, Canada; ^3^Integrated Program in Neuroscience, McGill University, Montreal, QC, Canada

**Keywords:** development, sex differences, corticolimbic circuit, perineuronal nets, amygdala, early life stress, fear, parvalbumin interneurons

## Abstract

The links between early life stress (ELS) and the emergence of psychopathology such as increased anxiety and depression are now well established, although the specific neurobiological and developmental mechanisms that translate ELS into poor health outcomes are still unclear. The consequences of ELS are complex because they depend on the form and severity of early stress, duration, and age of exposure as well as co-occurrence with other forms of physical or psychological trauma. The long term effects of ELS on the corticolimbic circuit underlying emotional and social behavior are particularly salient because ELS occurs during critical developmental periods in the establishment of this circuit, its local balance of inhibition:excitation and its connections with other neuronal pathways. Using examples drawn from the human and rodent literature, we review some of the consequences of ELS on the development of the corticolimbic circuit and how it might impact fear regulation in a sex- and hemispheric-dependent manner in both humans and rodents. We explore the effects of ELS on local inhibitory neurons and the formation of perineuronal nets (PNNs) that terminate critical periods of plasticity and promote the formation of stable local networks. Overall, the bulk of ELS studies report transient and/or long lasting alterations in both glutamatergic circuits and local inhibitory interneurons (INs) and their associated PNNs. Since the activity of INs plays a key role in the maturation of cortical regions and the formation of local field potentials, alterations in these INs triggered by ELS might critically participate in the development of psychiatric disorders in adulthood, including impaired fear extinction and anxiety behavior.

## Introduction

Numerous human epidemiological and observational studies have strongly linked adverse early life experiences with consequences on cognitive and emotional health (Bremne and Vermetten, 2001; Pechtel and Pizzagalli, 2011; Gould et al., 2012). Emotional, sexual and physical abuse, as well as neglect contribute to chronic early-life stress (ELS) in children, increasing their vulnerability to develop future psychiatric disorders, including anxiety, depression, and substance abuse (Weber et al., 2008; Green et al., 2010; Carr et al., 2013). It is estimated that 45% of childhood-onset mental health disorders and over 30% of later-onset of disorders are associated with early life adversity (Green et al., 2010; VanTieghem and Tottenham, 2017). The problem of childhood adversity is amplified because in humans as in non-human primates, there is a strong transgenerational transmission of infant maltreatment (Greene et al., 2020; Zeynel and Uzer, 2020). In macaques, stable transmission occurs mainly through the maternal line (Maestripieri, 2005; Morin et al., 2020) while in humans, both maternal and paternal early adversity can lead to adverse early experiences in their children. Adverse childhood experiences display marked sexual dimorphism in its occurrence, but also in the ensuing neurological and behavioral consequences which depend on the type, timing and duration of stress exposure (Fox et al., 2010; Gee and Casey, 2015; McLaughlin et al., 2019; White and Kaffman, 2019). For instance, girls are more likely to experience chronic sexual abuse, whereas boys are more likely to be exposed to physical violence and neglect (Maikovich-Fong and Jaffee, 2010; Gauthier-Duchesne et al., 2017; Coelho et al., 2018). Specific changes in gray matter volume (GMV) have been observed depending on the type of abuse encountered, with parental verbal abuse being associated with higher GMV in the adult auditory cortex and observations of decreased GMV in left and right visual cortex (V1) as well as cortical thinning of somatosensory cortex representing the genital areas in children exposed to sexual abuse (Teicher et al., 2016).

The heightened neuronal plasticity of the immature brain is usually advantageous and adaptive, allowing an individual to cope with and adapt to unfavorable conditions. However, this system may also become maladaptive in the face of early life adversity, dependent on genetic differences between individuals, and epigenetic changes of specific genes (McEwen, 2006; Buss et al., 2012; Franklin et al., 2012; Dunn et al., 2019). An alternative view is that ELS might selectively modify the way by which the brain processes information in a meaningful manner, leading to experience-dependent selective adaptations (Teicher et al., 2016). Structural and functional modifications would be geared toward ensuring increased survival and faster reaching of reproductive capabilities (Colich et al., 2020) in unfavorable conditions. This might come to the expense of accelerated aging since a recent report demonstrated that early life trauma predicted accelerated aging based on epigenetic clocks even though the trauma was not associated with age of menarche in this particular study (Hamlat et al., 2021). Childhood abuse, but not neglect, predicted faster epigenetic aging, emphasizing differences between the type of early adversity encountered. Similarly, a systematic review revealed that associations between early adversity and accelerated cortical thinning were region-and adversity specific, with threat-related adversity consistently associated with thinning in the ventromedial prefrontal cortex (vmPFC) (infralimbic, IL portion), and deprivation/neglect-type of adversity being associated with thinning in frontoparietal, default, and visual networks (Colich et al., 2020). Accelerated maturation of an adult-like type of corticolimbic connectivity has also been documented in individuals subjected to early life adversity (Gee et al., 2013a) even though a consistent association of ELS with amygdala-PFC connectivity was not detected in a recent large meta-analysis (Colich et al., 2020).

**FIGURE 1:**
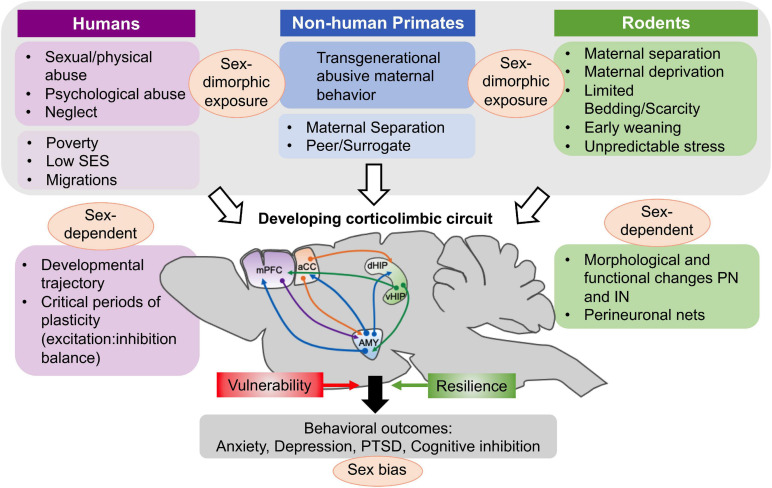
Factors associated with early life adversity in humans and experimental procedures in non-human primate and rodent studies that have been used in studies of ELS and adversity. Several, but not all of these factors and procedures produce a sexually dimorphic exposure in the offspring. In humans, poverty, being raised in a low SES milieu or being part of migratory movements might be less directly associated with sex-differences in exposure. These modalities causing ELS have in common that they occur during a critical developmental period for the corticolimbic circuit, affecting the normal developmental trajectory as well as critical periods of plasticity characterized by changes in the balance of excitation and inhibition in amygdala (AMY), medial prefrontal cortex (mPFC), anterior cingulate cortex (ACC), and hippocampus (dorsal and ventral). At the cellular level, both morphological and functional changes in principal neurons (PN) and inhibitory interneurons (IN) have been documented after ELS and these vary as a function of the sex of the offspring. The important role of perineuronal nets (PNNs) in stabilizing synaptic inputs on interneurons and closing critical periods of plasticity is also modified by ELS. Based on anatomical, morphological and functional changes triggered by ELS, behavioral outcomes of ELS and childhood adversity are further modified by genetic risk factors and epigenetic changes that confer risk and resilience to the offspring. In all three species examined in this paper, behavioral consequences of early adversity are differentiated according to sex and specific behavioral categories. Figure adapted from Murthy and Gould (2020).

Current research efforts in humans and preclinical models are largely dedicated to understanding how early life perturbations begin to shape later physiological and behavioral responses in a sex-dependent manner (Figure 1) and to find specific windows of vulnerability amenable to successful interventions (Callaghan et al., 2014; Walker et al., 2017; Luby et al., 2020). The corticolimbic circuit including the amygdala (and in particular the basolateral amygdala, BLA), medial prefrontal cortex (mPFC), and ventral hippocampus (vHipp) is critically implicated in ELS-induced psychopathologies and more widely in the generation of symptoms of depressive and anxiety disorders (Shin and Liberzon, 2010; Burghy et al., 2012; Godsil et al., 2013; Gee and Casey, 2015; Yan et al., 2017). Dysfunction in the corticolimbic circuitry also predicts increased fear responses, stress hypersensitivity and negative behavioral outcomes.

Central to this circuit is the amygdala and in particular, the BLA nucleus and its efferent output projections. The amygdala is as heterogeneous functionally as it is anatomically; it plays a complex role in emotional and social behavior (LeDoux, 2000; Adolphs, 2010), stress response (Roozendaal et al., 2009), learning and memory, anxiety (Rauch et al., 2003), and conditioning to appetitive or aversive stimuli (Shabel and Janak, 2009). In humans, ELS enhances amygdala reactivity during the presentation of negative emotional stimuli and weakens amygdala-prefrontal cortex (PFC) resting-state functional MRI (rs-fMRI) connectivity (Tottenham et al., 2011; Burghy et al., 2012; Pagliaccio et al., 2015). While connectivity between corticolimbic regions is established mainly through reciprocal glutamatergic projections involving principal pyramidal neurons, local inhibitory interneurons (INs) in each of these structures are critical in regulating the activity and function of principal neurons (PN) and ultimately regulate behavior. In addition, GABAergic INs are important for the assembly of neuronal circuits during critical developmental periods and they have been strongly associated with the development of psychopathologies (Marín, 2016). Interestingly, a subpopulation of local inhibitory neurons expressing parvalbumin (PV) is ensheathed by perineuronal nets (PNNs), which are proteoglycan rich assemblies derived from the extracellular matrix that control the opening and closure of critical windows of plasticity in several brain regions (Hensch, 2003, 2005). Experimental dissolution of PNNs within the corticolimbic circuit has allowed to unravel a critical role of these molecular assemblies in the formation and retrieval of adult fear memory (Ehrlich et al., 2009; Gogolla et al., 2009). Emerging studies are now also documenting that ELS significantly alters PNNs and their role of stabilizing synaptic inputs onto developing inhibitory INs. It is therefore conceivable that preclinical research on the activity of INs and their associated PNN structures after ELS will greatly enhance our knowledge of how the corticolimbic circuit develops in unfavorable conditions. Furthermore, it might allow for the specific targeting of IN activity in the design of novel therapeutic strategies.

In this paper, we will first review developmental aspects of the corticolimbic circuit and how these can be altered by ELS. We will then focus on some of the molecular and cellular changes observed in the amygdala and PFC that impact both principal neurons and inhibitory tone, and discuss how corticolimbic responses to ELS are impacted by sex and hemispheric differences.

## Amygdala-Prefrontal Cortex Development in Humans and Rodents

### Amygdala Development

Studies across species have revealed that amygdala development is protracted, with many changes in morphology and function of the BLA occurring postnatally in both humans and rodents. Thus, environmental insults, such as ELS, during critical periods of amygdala neurodevelopment could lead to deviations in the normal maturational trajectory and subsequently affect the integrity of the amygdala. The human amygdala, including the basolateral complex, emerges early in embryonic life (Muller et al., 2006) and is well established at birth (Avino et al., 2018). A structural MRI study reported that the amygdala undergoes a 40% increase in volume between 8 and 18 years old in neurotypical individuals (Schumann et al., 2004; Uematsu et al., 2012). The dramatic increase in amygdala volume from youth to early adulthood is due to several factors, including an increase in the number of mature neurons, as well as increased synaptogenesis and dendritic growth during neuronal maturation. Remarkably, the increase in the number of mature neurons in the entire amygdala is primarily driven by increases in the number of BLA neurons (30%), and less so by neuron increases in other amygdala nuclei such as the LA, which only exhibit a 3% increase in mature neuronal counts throughout development (Avino et al., 2018). Although adult neurogenesis has not been demonstrated in the adult primate amygdala, a region located near the temporal lobe lateral ventricle and adjacent to the BLA called the paralaminar nucleus (PL), does, however, contain a large population of neurons exhibiting an immature phenotype in adolescence and adulthood (Sorrells et al., 2019). The PL in the primate amygdala is similar in location to the dense clusters of GABAergic cells found around the BLA in the rodent amygdala, called the intercalated nuclei. The observation of a protracted maturation of excitatory neurons in the human amygdala and the fact that the most substantial changes in the composition of the PL occur during adolescence suggests that these neurons might remain highly vulnerable to the effects of ELS. Indeed, a recent study conducted in the macaque found that early life maternal separation modifies gene expression in the PL later in life (de Campo et al., 2017) and that neonatal hippocampal lesions accelerated the maturation of the PL neurons (Chareyron et al., 2016).

As in humans, the rat BLA forms in embryonic development (Berdel et al., 1997) since most neurons are generated between embryonic day (E) E14 and E17 (Bayer, 1980) and the total volume of the BLA increases until the third postnatal week (Rubinow and Juraska, 2009; Chareyron et al., 2011). Total neuron and glia numbers are unchanged from postnatal day (PND) 20 to PND35, but decrease by 13% between PND35-90 (Rubinow and Juraska, 2009). After PND35 and until adulthood, total BLA volume remains stable, but overall amygdala volume in female mice is generally smaller than that of males between PND30-90 (Koshibu et al., 2004). Principal BLA neurons undergo dramatic structural changes, particularly during the first postnatal month (Ryan et al., 2016). For instance, neuron soma size nearly doubles between PND7-28 and total dendritic length increases by three-fold until PND21 (Ryan et al., 2016). Dendritic arborization and spine density only reach maturity by PND28, which is comparable in age to early adolescence in humans (Quinn, 2005). Synaptogenesis in the BLA, as detected by presynaptic synaptophysin immunoreactivity, peaks at PND14 and stabilizes by PND30 (Morys et al., 1998). Increases in total synapse number during postnatal development may reflect the maturation of inputs to the BLA, particularly those coming from the mPFC which develop between PND13-21 (Bouwmeester et al., 2002b; Arruda-Carvalho et al., 2017).

Many of the morphological changes occurring in the rat BLA during postnatal development coincide with alterations in neuron physiology and synaptic plasticity. Passive membrane properties of principal BLA neurons, such as input resistance and membrane time constant, decrease with age and reach maturity at the end of the first postnatal month, in parallel with their structural development (Ehrlich et al., 2012). With regards to developmental changes in BLA synaptic plasticity, early studies have shown that high-frequency stimulation of LA inputs to the BLA results in modest long-term potentiation (LTP) on PND7-10, whereas after that and until PND19, long-term depression (LTD) is predominant (Thompson et al., 2008). The LTD state can be reverted to immature LTP via the application of GABA_A_ receptor antagonists, suggesting that developmental changes in GABAergic transmission likely modulate synaptic plasticity in the neonatal BLA (Thompson et al., 2008). Indeed, many aspects of GABA transmission in the BLA only mature at the end of the first postnatal month (Ehrlich et al., 2013). However, in contrast to earlier findings, successful LTP in the BLA was demonstrated as early as PND20 in male and female rats without any pharmacological blockade (Bender et al., 2017). Around the end of the second postnatal week in the BLA (Ehrlich et al., 2013), there is a fundamental switch of GABAergic transmission from depolarizing to hyperpolarizing that has also been documented in the mPFC and hippocampus (Ganguly et al., 2001; Le Magueresse and Monyer, 2013). This early excitatory GABA function might thus facilitate weak LTP in the young BLA in the absence of GABA_A_ receptor antagonists (Thompson et al., 2008).

### Amygdala-Prefrontal Cortex Circuit Development

A rich body of human and rodent literature has identified robust structural and functional connections between the amygdala and PFC during the developmental period (Bouwmeester et al., 2002a,b; Kim and Whalen, 2009; Gee et al., 2013a,b). In humans, the developmental trajectory of amygdala-PFC circuitry is hierarchical in nature, because the amygdala displays early functionality relative to the PFC (Tottenham and Gabard-Durnam, 2017). Functional magnetic resonance imaging (fMRI) data have shown that the amygdala in children (ages 4–9) exhibits strong reactivity to emotional stimuli (i.e., fearful faces) before mature connections with the PFC are established (Gee et al., 2013b). Furthermore, functional connectivity in response to fear between the amygdala and PFC switches from positive in early childhood to negative at age 10, and then becomes progressively more negative into young adulthood (ages 18–22) (Gee et al., 2013b). The exact nature of these reciprocal connections remains unclear, although some studies have suggested that negative amygdala-PFC functional connectivity in adolescence and adulthood results from the development of active inhibitory PFC influence on the amygdala (Hariri et al., 2003; Kim et al., 2003; Hare et al., 2008). Thus, it is conceivable that early amygdala activity is able to instruct development of the PFC. As the PFC matures, top–down signaling increases with age and likely contributes to the observed valence switch in connectivity (Gee et al., 2013b; Tottenham and Gabard-Durnam, 2017).

In neonatal rats (PND10-12), the onset of fear learning relies on the postnatal maturation of anatomical connections between the BLA and mPFC (Raineki et al., 2010; Tallot et al., 2016), which is predominantly achieved in the second and third postnatal weeks (Bouwmeester et al., 2002b; Arruda-Carvalho et al., 2017). Specifically, BLA efferents to the IL and prelimbic (PL) regions of the mPFC emerge between PND7-9 and reach an adult-like innervation pattern in the mPFC by PND13. Most of the BLA-mPFC projections discovered in preweaning (PND18-20) rats are intra-hemispheric, but few inter-hemispheric connections also exist at this age (Verwer et al., 1996). The number of BLA to PFC projections continues to increase throughout adolescence and only stabilizes in adulthood (Cunningham et al., 2002). The descending mPFC to BLA projections mature later, between PND13–21 (Bouwmeester et al., 2002b) and remain relatively stable until they undergo pruning in late adolescence (PND45) (Cressman et al., 2010). Thus, as described previously in humans (Tottenham and Gabard-Durnam, 2017), there is a sequential type of maturation between the ascending and top–down components of this bidirectional circuitry that influences the development of behavioral responses to fear, fear extinction and fear memory.

### Development of Amygdala-Related Fear Behaviors in Rodents

Most of our understanding of the corticolimbic fear circuitry has emerged from seminal studies using Pavlovian conditioning, an experimental paradigm that is frequently implemented to examine fear learning in both humans and rodents (Fanselow and LeDoux, 1999; Maren, 2001; Carrere and Alexandre, 2015). Fear expression may be decreased through fear extinction, which is considered a different learning process (Krabbe et al., 2018) that occurs in three phases known as acquisition, consolidation and retrieval (Quirk and Mueller, 2008; Lee et al., 2015). Once the fear memory is consolidated, it may be reinstated by presenting the conditioning stimulus (CS) in the same context in which extinction occurred (Quirk and Mueller, 2008) or renewed by placing animals in a novel context (Marschner et al., 2008; Maren et al., 2013). Importantly, the renewal of context-dependent fear memories is developmentally regulated in rats, since extinguished fear responses can only be reinstated after the third postnatal week (Kim and Richardson, 2007). Before this time, fear experience is erased and there is no fear recall when rats are placed in a novel context (Kim and Richardson, 2007).

The protracted postnatal development of the corticolimbic circuit contributes to changes in the maturation of emotional behavior (Ryan et al., 2016; Sullivan and Opendak, 2020a). During the first week and a half of postnatal life, rat pups have limited hearing and vision and quickly learn to preferentially recognize maternal odors which promotes caregiver attachment (Sullivan, 2001; Tallot et al., 2016; Sullivan and Opendak, 2020b). Many studies using conditioning paradigms in neonatal rodents have therefore used olfactory cues as the CS (Jovanovic et al., 2013). Elegant studies have shown that during the first 2 weeks of postnatal life in rats, the mother has the ability to modify the emotional valence of stimuli (Moriceau and Sullivan, 2006; Santiago et al., 2017; Sullivan, 2017) even though pups can learn aversive responses after PND10. Before that time, PND8 rats are able to pair an odor and a foot shock (US), but the conditioned response is approach rather than avoidance to the shock (Sullivan et al., 2000). Once pups leave the nest around PND10, this same odor-shock conditioning can produce avoidance responses (Moriceau et al., 2006; Thompson et al., 2008), although when conditioning occurs in the presence of the mother, approach rather than avoidant responses to the CS are observed. When pups grow in the presence of an abusive mother, the beneficial buffering effect of the mother is lost and early avoidance responses to shock are observed. Other aspects of emotional responses such as freezing and fear-potentiated startle also mature along the development of the corticolimbic circuit. Conditioned freezing in rats emerges on PND16-18 (Hunt et al., 1998; Barnet and Hunt, 2006; Ryan et al., 2016) and reaches adult-like levels by PND23-27 (Jovanovic et al., 2013). Similarly, fear-potentiated startle to a tone is observed after weaning on PND23, but not earlier in the rat (Hunt et al., 1994).

Fear extinction outcomes and cellular mechanisms across development are also fundamentally different. In neonates (PND16), extinction training promotes the permanent erasure of fear memories (Kim and Richardson, 2007, 2008). By contrast, the same protocol in PND23 rats reinstates extinguished fear, as seen in adulthood (Kim and Richardson, 2007, 2008, 2010). This switch appears to be mediated by developmentally regulated changes in NMDAR activity in the brain (Langton et al., 2007), as their blockade only disrupts fear extinction on PND23, while PND16 extinction is unaffected. PNNs that surround primarily GABAergic neurons may also play a significant role in protecting fear memories from erasure at later developmental stages because enzymatic “dissolution” of PNNs in the BLA of adult rats reinstated the phenotype of erasure of fear memory that is normally only observed in young neonatal rats (Gogolla et al., 2009). Lastly, inactivation of the amygdala [BLA, lateral amygdala (LA), central amygdala (CeA)] disrupts extinction on PND17 but not on PND24 (Kim and Richardson, 2008), suggesting that extinction becomes more dependent on the mPFC once reciprocal connections between the BLA and mPFC are established (Kim and Richardson, 2008; Sotres-Bayon and Quirk, 2010; Jovanovic et al., 2013).

### Summary

In both humans and rodents, development of the corticolimbic circuit is not achieved until the juvenile, peri-adolescent period, with ascending projections from the amygdala to the prefrontal cortex maturing earlier than top–down control of the amygdala by the PFC. The progressive change in connectivity between these two regions, follows important maturation processes in the amygdala with large increases in volume, synaptogenesis and dendritic arborization occurring up to the third week of life in rodents and 18 years in humans. The protracted and predominantly postnatal development of the corticolimbic circuit in human and rodents makes this circuit particularly sensitive to environmental stressors occurring during the neonatal and juvenile periods (Bouwmeester et al., 2002a,b; Gee et al., 2013a; Tottenham and Gabard-Durnam, 2017). As a consequence, the acquisition of fear can be accelerated in adverse conditions and in particular, negative, more “mature” connectivity between the amygdala and the PFC is observed in children raised in institutions compared to those raised in their biological families (Gee et al., 2013a). Similarly, the perception of an aversive situation is observed shortly after the first week in rodents, although it is usually buffered by the presence of the mother. In the case of an abusive mother, the buffering effect is lost and rat pups exhibit enhanced aversive learning. Conditioned freezing to an aversive shock is learned by the 2nd or 3rd week of life in rodents, although fear memory only appears after weaning in parallel with the maturation of top down projections from the PFC and amygdala that are responsible for fear extinction. Because of the sequential maturation of the ascending (fear acquisition) and descending (fear extinction) projections in both human and rodents and the sensitivity of the maturing amygdala to stress, it is understandable that exposure to early life stress and trauma has such severe consequences on the exaggerated processing of fear and sustained fear memory that ultimately leads to an anxiety phenotype.

## Impact of Early Life Stress on the Corticolimbic Circuit

Early adversity produces robust changes in the amygdala and amygdala-prefrontal pathway across species that associate with emotional difficulties throughout the life-span (Ellis et al., 2004; Burghy et al., 2012; Gee and Casey, 2015; Hanson et al., 2015). Major changes are observed in the structure and excitability of the amygdala as well as in the functional connectivity between the amygdala and the PFC. These changes are long-lasting and might well represent the neural underpinnings of anxiety and mood disorders.

### Structural Alterations in the Amygdala Following Early Life Stress

The amygdala is one of the few structures that generally increases in volume in response to chronic stress (Vyas et al., 2002; Mehta et al., 2009; Tottenham et al., 2010) and this might be related to the high density of glucocorticoid receptors present in the amygdala (Geuze et al., 2012). The amygdala is extremely sensitive to the effects of both acute (Mitra et al., 2005; Rodriguez Manzanares et al., 2005; Duvarci and Pare, 2014; Kim et al., 2014) and chronic stress exposure (Vyas et al., 2002, 2004, 2006; Tottenham et al., 2010; Rau et al., 2015). In humans, somewhat conflicting results on the effects of early life adversity on amygdala volume have been reported depending on the developmental timing of stress exposure, as well as the duration and intensity of the stress (Lupien et al., 2009; Fox et al., 2010; Gee and Casey, 2015). For instance, children reared in orphanages display significantly larger bilateral amygdala volumes (Mehta et al., 2009; Tottenham et al., 2010), while children who suffered from physical abuse or early neglect were found to have significantly smaller left amygdala volumes (Hanson et al., 2015). Similarly, children exposed to maternal depressive symptomology since birth (i.e., prolonged stress) exhibit increased amygdala volume, while hippocampal volume remains unaffected (Lupien et al., 2011). Interestingly, a recent study found that self-reported childhood neglect was associated with sex-specific and lateralized changes in the adolescent amygdala, with boys, but not girls, having larger right amygdala volumes (Roth et al., 2018). In adulthood, greater exposure to chronic stressors during childhood, measured using an index of cumulative risk exposure, is positively correlated with enlarged amygdala volumes (Evans et al., 2016).

Consistent with many human studies, the rodent BLA also undergoes hypertrophy in response to chronic stress. Juvenile chronic restraint stress (PND20-41) increases BLA pyramidal neuron dendritic length in male and female rats (Eiland et al., 2012). Similarly, chronic immobilization stress (CIS, 2 h/day for 10 days) in adult rats enhances dendrite arborization of pyramidal and stellate neurons in the BLA (Vyas et al., 2002). Interestingly, the same stress paradigm does not impact neuron morphology in the CeA (Vyas et al., 2003). Chronic stress also alters neuron morphology in the hippocampus by reducing spine density and arborization, however these changes are reversible, unlike those observed in the BLA (Vyas et al., 2003, 2004). Even after animals are given a 21-day stress-free period following CIS, the dendritic arbors in their BLA continue to increase in size, indicating that the amygdala may be ‘sensitized’ and thus more resistant to recovery following chronic stress (Vyas et al., 2004; Gee and Casey, 2015).

While the effects of chronic stress on the amygdala have been well documented in adults, less understood are the effects of chronic ELS on the structure of the neonatal and juvenile amygdala. Prenatal stress was shown to increase BLA volume, as well as neuron and glia density in male juvenile (PND25) rat offspring (Kraszpulski et al., 2006). Even though these structural differences appear to dissipate by adulthood, it is suggested that the accelerated BLA growth trajectory could impair developing reciprocal connections with the mPFC and enhance fear behaviors in the long-term (Kraszpulski et al., 2006). In our studies, we did not find significant volumetric changes in the preweaning amygdala after ELS, although there was a clear trend toward an increased volume (Guadagno et al., 2018a,b). ELS in the form of early weaning (on PND14) causes precocious myelination in the BLA of adolescent male mice (Ono et al., 2008). Collectively, these findings indicate that the consequences of ELS on the amygdala can be observed early as alterations in amygdala structural integrity in neonates, such as increased spine density that is observed exclusively in males (Guadagno et al., 2018a) occur in parallel with enhanced amygdala reactivity and neuron excitability (Guadagno et al., 2020).

### Effects of Early Life Stress on Amygdala Reactivity and Excitability

Humans with a history of ELS typically have larger amygdala volumes that presumably contribute to heightened amygdala reactivity to emotional stimuli (Tottenham et al., 2010). Retrospective (Dannlowski et al., 2013; Van Harmelen et al., 2013) and recent prospective studies (Javanbakht et al., 2015; Evans et al., 2016) of adults exposed to early adversity have identified exaggerated amygdala reactivity to negative emotional cues. In line with these findings, children and adolescents (all right-handed, males and females) that were previously institutionalized display right-lateralized hyperreactive amygdala responses to fear faces (Gee et al., 2013a). Youths having experienced caregiver deprivation or emotional neglect similarly display enhanced amygdala activation during the analysis of detailed emotional faces, but only on the left side (Maheu et al., 2010). In this case, left as opposed to right-lateralized amygdala activity may be due to the type of task used that engaged more extensive and conscious emotional processing (Maheu et al., 2010). Interestingly, the earlier the onset of childhood maltreatment, the more severe the hyperreactive amygdala responses to emotional stimuli, which further emphasizes the importance of the timing of postnatal ELS exposure in predicting amygdala outcomes (McCrory et al., 2013). It is worth noting that adverse rearing conditions increase amygdala reactivity to fearful relative to neutral faces in children (Tottenham et al., 2011), a response normally only observed in naïve adults (Thomas et al., 2001), suggesting that ELS might enable precocious amygdala development.

Amygdala reactivity in rodents is similarly enhanced following exposure to chronic stress during early life and adolescence (Raineki et al., 2012; Malter Cohen et al., 2013; Rau et al., 2015). More specifically, amygdala neural activity, measured with c-Fos immunohistochemistry, is increased after exposure to acute forced swim in adolescent male and female rats subjected to ELS (Raineki et al., 2012). ELS in the form of altered maternal care between PND2–21 also increases the expression of c-Fos protein in the BLA of juvenile and adolescent male mice (PND26–34) exposed to a threatening context (Malter Cohen et al., 2013). ELS-induced changes in the development of the amygdala might result from dysregulated HPA axis activity that is associated with ELS. For example, the emergence of amygdala fear reactivity is accelerated by increasing CORT levels systemically or by injecting CORT directly in the amygdala of male and female neonatal rats (Moriceau et al., 2006). Following adolescent social isolation, adult male rats show hyperexcitability of BLA pyramidal neurons (Rau et al., 2015). In summary, the few existing studies on the effects of ELS on the juvenile or adult amygdala emphasize amygdala hyperexcitability and increased emotional behavior. Increased synaptic plasticity found in the right BLA of juvenile male rats exposed previously to ELS (Guadagno et al., 2020) might constitute one of the mechanisms leading to amygdala hyperexcitability after ELS, seeing as chronic stress in adulthood increases LTP formation in the amygdala (Dalton et al., 2012; Suvrathan et al., 2014).

### Functional Amygdala-mPFC Connectivity Is Altered After Early Adversity

Studies in both rodents (Yan et al., 2017; Johnson et al., 2018; Guadagno et al., 2018b; White et al., 2020) and humans (Burghy et al., 2012; Gee et al., 2013a; Peverill et al., 2019) have demonstrated that functional connectivity within the corticolimbic circuitry is extremely sensitive to ELS exposure. In humans, early life adversity has been shown to weaken resting state amygdala-PFC functional connectivity in childhood (Gee et al., 2013b), adolescence (Burghy et al., 2012) and adulthood, suggesting that ELS exposure programs lasting changes in the functional integrity of the corticolimbic circuit. Based on results found in 7–8 year-old children, it was argued that heightened amygdala reactivity found in previously institutionalized youths accelerates the development of a more “mature” and weaker amygdala-PFC connectivity as assessed by fMRI in response to an emotional task (Gee et al., 2013b). However, a more recent study using a larger sample population has failed to replicate the pattern of changes seen in the study by Gee et al. (2013b), Zhang et al. (2019) and challenged the view that changes in the patterns of amygdala-mPFC connectivity with ELS truly reflects accelerated maturation of the corticolimbic circuit. Regardless of the notion of accelerated maturation, reduced functional connectivity between the amygdala and other brain structures important for emotional processing could explain the behavioral outcomes observed in the long term. In particular, reduced functional connectivity between the amygdala and anterior cingulate cortex (ACC) is also observed in children with high levels of prior cumulative stress exposure (Pagliaccio et al., 2015) and between the right amygdala and vmPFC in maltreated females, but not males (Herringa et al., 2013). Reduced connectivity between the vmPFC and left hippocampus was seen in both sexes (Herringa et al., 2013), emphasizing the fact that at least in humans, sex-related ELS effects exhibit hemispheric specificity that is dependent of the regional circuit considered. The reduced functional connectivity between the amygdala and several subregions of the prefrontal cortex was also observed in a longitudinal study in infant to juvenile macaques raised by abusive mothers (Morin et al., 2020). This effect was particularly observed in maltreated females and was partially predicted by increased exposure to cortisol in infancy.

Neuroimaging studies in rodents exposed to ELS have revealed equally disrupted patterns of amygdala functional connectivity (Holschneider et al., 2016; Yan et al., 2017) that persist until adulthood (White et al., 2020). For instance, exposure to unpredictable varied stressors (PND14–25) in mice increased amygdala-PFC, amygdala-vHipp (Johnson et al., 2018) and vHipp-PFC connectivity in adult male, but not female offspring (White et al., 2020). In this study, tractography revealed a significant hemispheric effect on amygdala-vHipp connectivity, with higher connectivity changes in the left hemisphere. When earlier time points were examined, early adversity in preweaning rats reduced rs-fMRI connectivity between the BLA and mPFC (Guadagno et al., 2018b) and this was also documented in adolescent and adult male rats (Yan et al., 2017). This suggests that there might be significant differences between ELS models, animal models (rat vs. mouse) or specific time windows associated with differential sex effects of ELS. Importantly, a recent study used high resolution *ex vivo* diffusion tensor imaging to visualize bilateral BLA-mPFC projections in PND56 rats and compare naïve to ELS-exposed rats. The results of this study showed an increased number of tracts crossing the midline (Bolton et al., 2018), indicating that ELS might cause aberrant inter-hemispheric structural, as well as functional connectivity.

Early life stress affects amygdala-related emotional behaviors, as indicated earlier, most studies on early adversity in children report an increased vulnerability to developing anxiety and mood disorders and it is thought that molecular, cellular and circuit-based modifications in the development of the corticolimbic circuit are essential mechanisms that promote such vulnerability. Examples are numerous. For instance, children reared in orphanages with greater amygdala volumes display more internalizing behaviors and anxiety (Tottenham et al., 2010), which are risk factors for the development of future psychopathologies (Hofstra et al., 2002). Indeed, roughly half of the children tested in this study met diagnostic criteria for at least one psychiatric illness, of which ∼20% were anxiety disorders (Tottenham et al., 2010). Higher levels of self-reported childhood neglect in adolescent males associate with enhanced right amygdala activity and elevated anxiety symptoms (Roth et al., 2018). Adults with anxiety disorders and a history of ELS also display enhanced amygdala activity to threat cues (Bremner et al., 2003a,b, 2005). It is believed that atypically large amygdala volumes in ELS-exposed individuals might allow for greater processing of negatively valenced information and increased amygdala sensitivity to fearful cues (Tottenham et al., 2010; Gee et al., 2013a). In addition, the heightened amygdala reactivity observed in early development might actually reduce the functional connectivity between the amygdala and the PFC (Burghy et al., 2012; Gee et al., 2013b), leading to a phenotype of higher trait anxiety and emotional reactivity (Burghy et al., 2012; VanTieghem and Tottenham, 2017). Of interest is the notion that ELS might modify the number of fiber tracts crossing the midline that target cortical regions, as well as modify their integrity (Bolton et al., 2018; Howell et al., 2019). This is important since previous reports have shown that the structural integrity of white matter tracts between the amygdala and the PFC is inversely correlated with anxiety symptoms in adult humans (Kim and Whalen, 2009; Burghy et al., 2012).

In rodents, exposure to chronic stress during the developmental period also has lasting effects on the male adolescent and adult amygdala, as evidenced by increased anxiety and conditioned fear responses in these animals (Stevenson et al., 2009; Eiland et al., 2012; Molet et al., 2014; Guadagno et al., 2018a). Adolescent male rats reared under suboptimal conditions display increased BLA neural activity and enhanced anxiety and depressive-like behaviors (Raineki et al., 2012). Importantly, when the amygdala is temporarily deactivated, depressive-like behaviors in ELS animals are reversed, suggesting a causal link between amygdala functioning after ELS and behavioral dysfunctions (Raineki et al., 2012). Similarly, enhanced BLA neural activity observed in juvenile and adolescent mice exposed to ELS increases fear responses to a threatening context (Malter Cohen et al., 2013). Hypertrophy of BLA neurons in male and female adolescent mice following chronic juvenile stress associates with enhanced anxiety-like behavior in the elevated plus maze (Eiland et al., 2012). In line with the human literature (Gee et al., 2013b), reduced rs-fMRI connectivity between the BLA and mPFC after ELS is linked to long-term decreases in social interactions and increased depressive behaviors in adult rats (Yan et al., 2017).

### Summary

In both human and rodents, exposure to early adversity increases amygdala volume and reactivity, in particular to fearful faces in children. The earlier the onset of maltreatment, the higher the magnitude of amygdala hyperactivity. Precocious myelination and increased synaptic plasticity has been observed in the rodent amygdala after early adversity, with males being more susceptible in general to the effects of early stress. In humans, it has been suggested that adversity might also enable precocious amygdala development. An accelerated maturation of the amygdala together with aberrant inter-hemispheric structural connectivity might precipitate the characteristic reduced functional connectivity of the amygdala with other brain regions demonstrated consistently across species. Most models of ELS exposure in humans, non-human primates and rodents have demonstrated that it creates a phenotype of enhanced anxiety and depression as well as reduced social behavior that is likely to be mediated, at least in part, by morphological and functional changes in the corticolimbic circuitry. Variations in the magnitude of the effects, the type of behavior mostly impaired and the possibility for resilience might depend on the genetic and epigenetic makeup of the individual as well as the timing and nature of the early stressors (Tottenham and Sheridan, 2009; Gee and Casey, 2015; McCarthy et al., 2017).

## Sex and Hemispheric Differences in Corticolimbic Development as Modulating Factors in the Effects of Early Life Stress

Sex differences in the development of the rodent corticolimbic circuit and its specific components has been recently elegantly reviewed (Premachandran et al., 2020) and sex effects have been outlined at the level of the amygdala, mPFC and hippocampus in terms of differences in volume, morphology, synaptic organization, cell proliferation, microglia, and GABAergic signaling. Morphological and functional changes over the course of development have been studied mostly in the dorsal hippocampus, reporting both transient, and long-term effects of sex on this structure. Interestingly, females undergo an earlier switch in GABA_A_-mediated excitation to inhibition during the first postnatal week in rodents, occurring between PND4–7 compared to PND8–14 in males (Premachandran et al., 2020). This difference in the timing of inhibition onset might be critical to determine further developmental processes in this structure and to identify potential windows of sensitivity to external stimuli. Consequences of sex-differences in maturation of the hippocampus have been found in the context of behaviors that are mediated by the dorsal hippocampus such as cognitive strategies for spatial learning (Shansky, 2018; Grissom et al., 2019). In contrast, few studies have investigated sex differences in the development of the vHipp regions and results of sex-related anxiety studies early in development have been somewhat contradictory.

The rodent amygdala, especially the BLA, has been the subject of far fewer studies examining sex and hemispheric differences during development. Work focusing on the medial amygdala (MeA), which is the region of the amygdala containing the highest concentration of estrogen and androgen receptors, has suggested that this amygdala subregion matures faster than other subregions in both sexes (Chareyron et al., 2011). Volumes of the MeA reach adult-like values faster in females (PND5) than in males (PND21) males, although increased total structural volumes, dendritic volumes and neuron numbers were observed in prepubertal (PND26–29) male compared to female rats (Cooke et al., 2007), independent of amygdala side. They also found that MeA neurons in males had more frequent miniature excitatory postsynaptic currents (mEPSCs) and a greater number of excitatory synapses on dendritic spines compared to females, again only on the left side (Cooke and Woolley, 2005). Enhanced MeA neuron numbers in males persist until adulthood (PND60) (Morris et al., 2008). For the developing rat BLA, no sex differences or interactions with amygdala hemisphere were found for total volume or number of neurons on PND20, 35, or 90 (Rubinow et al., 2009; Guadagno et al., 2018b), although exposure to ELS increased spine density in the BLA of males, but not female PND10 and PND21 rats (Guadagno et al., 2018a). There was also no significant effect of sex on maturation of physiological properties of BLA pyramidal neurons in the first postnatal month (between PND7–28) (Ehrlich et al., 2012). Only in adulthood do some sex and hemispheric differences emerge in the BLA under normal circumstances. For instance, male adult rats have more dendritic spines on pyramidal BLA neurons compared to females (Rubinow et al., 2009), and greater numbers of PV-positive cells in the left compared to the right BLA (Butler et al., 2018). Thus, effects of sex and laterality in the rat emerge earlier in the developing MeA than in the BLA, highlighting that these amygdala nuclei are fundamentally different, both anatomically and functionally (Sah et al., 2003). If there are little underlying sex or laterality differences during the normal BLA developmental trajectory, sex and hemispheric-dependent effects of ELS on the young rat BLA (Guadagno et al., 2020) could likely be a direct consequence of the stress exposure.

In the human amygdala, a structural MRI study found that between 1 month to 25 years of age, the total growth period for males was longer than that of females, and likely contributed to the observed larger male amygdala volume (Uematsu et al., 2012). However, growth of the amygdala is not linear because the dramatic gains in volume at 5 and 6 years of age become more moderate as youths enter early adolescence with the volumetric growth of the left amygdala peaking earlier than the right amygdala (Russell et al., 2021). There is some discrepancy about sex differences in amygdala growth, some reports showing that the female amygdala volume (including white and gray matter) peak in pre-adolescence, 18 months earlier than in males, regardless of hemisphere (Uematsu et al., 2012) while others found that amygdala GMV is increased in males compared to females (ages 8–15), only on the left side, and varies according to pubertal stage and circulating testosterone levels (Neufang et al., 2009). These findings suggest that gonadal steroid levels might play an important role in governing sexually dimorphic amygdala development. Hemispheric differences in total amygdala volume are seen in males during development, but the direction of this asymmetry is related to the age range and type of amygdala tissue imaged. For instance, in contrast to results reported by Neufang et al. (2009), right asymmetry of amygdala volume was observed in males, but not females, from infancy until adulthood (Uematsu et al., 2012) and it is believed that the right and left amygdala develop at different rates and may be involved in different emotional regulation processes (Wager et al., 2003; Uematsu et al., 2012; Wen et al., 2017). Fetal testosterone has been shown to positively correlate with GMV in the left, but not the right, amygdala of typically developing boys (aged 8–11 years old) (Lombardo et al., 2012), suggesting that early organizing effects of sex hormones prenatally may contribute to lateralized amygdala volume. Other environmental factors in early preterm infants like exposure to musicotherapy in the NICU have been found to improve white matter maturation and increase amygdala volume compared to preterm infants with standard of care (Sa de Almeida et al., 2020).

While maturation of the hippocampus and amygdala occurs relatively early in development, that of the mPFC is not complete until young adulthood in humans, non-human primates and rodents (Cunningham et al., 2002). In the rat, there appear to be waves of development/pruning of the mPFC, in particular in the PL with a peak of volume around PND14 and PND24 and a gradual reduction in volume by PND35 (Van Eden and Uylings, 1985) associated with neuronal loss, which continues until adulthood (Markham et al., 2007). The neuronal decline is more pronounced in females, which exhibit a sharp decline around puberty onset (PND35–45) while in males, the neuron loss continues more linearly between periadolescence and adulthood (Willing and Juraska, 2015). Hemispheric mPFC differences have been documented in the adult rat, in particular for dopaminergic innervation and the role of the mPFC in neuroendocrine stress responses (Sullivan and Gratton, 2002), however, these studies have not examined the onset of mPFC laterality during development. Basal extracellular Dopamine concentrations, but not 5HT or norepinephrine exhibited sex-related lateralized differences in the vmPFC of adolescent rats, suggesting that differential maturation of neurotransmitter innervation of mPFC could participate in lateralized mPFC function already in adolescence (Staiti et al., 2011). Exposure of neonatal rats to ELS in the form of early weaning and unpredictable adolescent stress showed that the mean excitatory latency of vmPFC neurons to *in vivo* stimulation of the amygdala was longer in ELS compared to control rats in the right compared to the left hemisphere (Ishikawa et al., 2015), supporting impaired connectivity that could be more pronounced in the right vs. left vmPFC.

A large number of studies have found that neuronal, functional and behavioral outcomes of ELS are sexually dimorphic (Figure 1) and recent reviews have addressed this topic (Loi et al., 2014; Walker et al., 2017; White and Kaffman, 2019; Bath, 2020). In several experimental models of ELS, adult female behavior appears to be significantly less impaired than male behavior, but this should not be interpreted to indicate that females are entirely resilient to early adversity. In fact, many human studies have shown that females exposed to childhood maltreatment are more sensitive (White and Kaffman, 2019) and likely to develop stress-related mental disorders, such as anxiety and depression, compared to males (McCarthy, 2016; McCarthy et al., 2017). Other reports have specified that sex differences in the behavioral consequences of early adversity strongly depend upon genetic susceptibility and the type and developmental timing of maltreatment (Walker et al., 2017; White and Kaffman, 2019). In addition to being caused by chromosomal, epigenetic and hormonal differences in neurodevelopmental events, sexually dimorphic outcomes can also be due to differences in maternal care toward males and females after exposure to ELS as outlined in a review by Bath (2020). In the case of the development of the corticolimbic system, it is still unclear whether ELS affects the normal sexually dimorphic maturation of corticolimbic brain regions at different time points of development in male and female individuals or whether ELS affects maturational processes differentially between males and females.

## Early Life Stress Modulates Local Amygdala Circuits and Inhibitory Interneurons

When considering studies on the mechanisms of fear learning, extinction and fear memory, the large majority of studies have mainly considered the role of principal excitatory projection neurons (PN) because they exhibit varied response properties that are input-selective and they are highly interconnected within several brain networks. PNs constitute the large majority of cells in areas of the corticolimbic circuit, and in particular, in the BLA and PFC, and their critical role in emotional processing has been demonstrated. However, there is mounting evidence to suggest that their activity is highly orchestrated by their interactions with local inhibitory, GABAergic INs (Lucas and Clem, 2018). Inhibitory GABA INs are diverse in many aspects such as their pattern of peptidergic co-expression, their axonal targets, firing characteristics and dendritic morphology. They can actively shape network activity and this is particularly relevant during the developmental period when both PNs and INs mature at different rates.

### Local Amygdala Circuits in Fear Regulation

**FIGURE 2:**
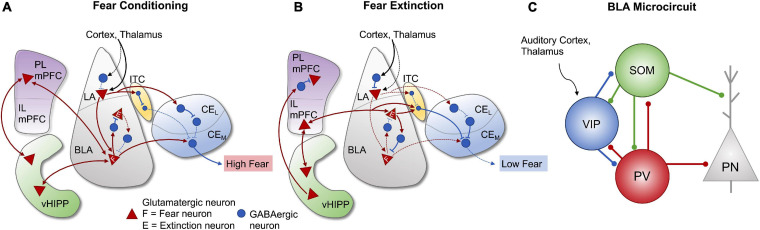
Amygdala and corticolimbic pathways involved in fear conditioning and extinction. Strengthened and weakened connections are represented by the solid and dashed lines, respectively. **(A)** During fear conditioning, multimodal sensory information from the cortex and thalamus primarily converges onto lateral amygdala (LA) projection neurons and activates descending projections to fear neurons in the BLA, as well as inputs to GABAergic neurons in the intercalated cell masses (ITC). BLA fear neurons are also stimulated by projections from the ventral hippocampus (vHIPP) and prelimbic medial prefrontal cortex (PL mPFC). Output neurons in the medial division (Ce_M_) of the CeA are disinhibited by GABAergic neurons in the ITC and lateral division of the central amygdala (Ce_L_) and activated by BLA fear neurons, leading to high fear responses. **(B)** During fear extinction, incoming sensory information mostly converges onto LA inhibitory interneurons, leading to suppressed activity of LA projection neurons and decreased BLA fear neuron activity. Projections from the vHIPP inhibit PL mPFC activity and stimulate infralimbic mPFC activity (IL mPFC). The IL region activates glutamatergic extinction neurons in the BLA, which stimulate neurons in the ITC and inhibit BLA fear neurons, thus resulting in the inhibition of Ce_M_ output neurons and reduced fear responses. **(C)** Organized microcircuit of feedforward inhibition in the rodent BLA regulate the activity of principal neurons (PN) under associative fear learning. VIP interneurons receiving direct inputs from the auditory cortex and thalamus are strongly activated by unexpected aversive events (US: aversive shock) and they gate information flow to a large fraction of PNs by releasing them from the strong inhibition provided by SOM and PV interneurons. There is an extended reciprocal inhibitory connectivity between all three interneuron subtypes found in the BLA. Figure adapted from Ehrlich et al. (2009), Sotres-Bayon and Quirk (2010), Sotres-Bayon et al. (2012), Lee et al. (2013), Duvarci and Pare (2014), Arruda-Carvalho and Clem (2015), Krabbe et al. (2019), and Zimmermann et al. (2019).

During acquisition and consolidation of conditioned fear, multimodal sensory information from the thalamus and cortex converges onto projection and GABAergic neurons of the LA, but also directly to BLA neurons (Romanski and LeDoux, 1993; Ehrlich et al., 2009; Duvarci and Pare, 2014). Activated LA projections terminate on glutamatergic cells in the BLA (Ehrlich et al., 2009), which engage separate disinhibitory mechanisms involving PV-positive and somatostatin-positive INs (Wolff et al., 2014) to facilitate excitatory output to the CeA. During the conditioned stimulus (CS = auditory tone), PV-expressing cells are stimulated and inhibit somatostatin INs, which results in dendritic disinhibition of BLA PNs (Wolff et al., 2014). Conversely, presentation of the unconditioned stimulus (US = footshock) inhibits both PV and somatostatin INs, through the action of vasoactive intestinal peptide (VIP)-positive INs on both PV and somatostatin neurons. This process of sequential feedforward inhibition ultimately leads to the disinhibition of BLA PNs, activation of CeA output neurons and enhanced fear learning (Ehrlich et al., 2009; Duvarci et al., 2011; Tovote et al., 2015; Krabbe et al., 2019; Figure 2). In addition to processing and relaying sensory information to the CeA, the BLA encodes (Gale et al., 2004) and stores permanent fear memories (Fanselow and LeDoux, 1999; Maren, 2001; Repa et al., 2001; Gale et al., 2004), a process that is dependent upon connections with the PL mPFC and vHipp (Arruda-Carvalho and Clem, 2014). Disruption of BLA activity not only impairs fear acquisition and conditioned fear responses (Amano et al., 2011), but also fear extinction (Herry et al., 2006). It is now recognized that within the BLA, functionally distinct populations of neurons encode fear acquisition (fear neurons) and fear extinction (extinction neurons). As illustrated in Figure 2, these neurons are differentially connected with the mPFC and vHIPP, in particular, to regulate many aspects of fear processing (Herry et al., 2008). Taken together, these data demonstrate that acquisition, extinction and memory of fear require precisely coordinated interactions between PNs and several subtypes of local inhibitory INs. Stress-induced disruption in the establishment and maturation of any of these local circuits is likely to have lasting consequences on fear processing and the storage of fear memories. Understanding how these local amygdala inhibitory circuits are established and where critical elements of regulation lie will help clarify some of the mechanisms through which, early life adversity dysregulates emotional behavior.

### Amygdala Inhibitory Interneurons

Approximately 80–85% of the neurons in the BLA are glutamatergic PNs with spiny dendrites (McDonald, 1982) and their activity is coordinated by interactions with local INs, which constitute the remaining 20% of BLA neurons. PNs densely innervate the dendritic spines of other surrounding PNs, as well as the peri-somatic and distal dendrites of spine-sparse GABAergic INs (Muller et al., 2006). Reciprocally, most of the glutamatergic PNs within the BLA are the post-synaptic targets of INs, which can significantly influence their activity (Spampanato et al., 2011). The INs in the BLA are characterized by their expression of calcium binding proteins (calbindin, calretinin, parvalbumin) and/or neuropeptides like somatostatin, CCK or VIP (Muller et al., 2006; Spampanato et al., 2011) and their pattern of innervation of PNs (Muller et al., 2007; Woodruff and Sah, 2007). For instance, PV-positive cells with basket-type morphology synapse on the peri-somatic region of PNs, including the soma, axon initial segment, and proximal dendrites, whereas those with ‘chandelier’ morphology form another class of axo-axonic synapses, innervating the largest portions of the axon initial segment (Gabbott et al., 2006). In addition, PV-INs that constitute half of the BLA INs form synapses with INs from their own or other groups (Spampanato et al., 2011). They are not only critical in generating theta-range oscillations (30–80 Hz) (Marín, 2012), but are also differentially recruited according to the specific stimuli associated with fear conditioning and inhibition (Courtin et al., 2014; Wolff et al., 2014; Krabbe et al., 2019). IN firing properties are incredibly diverse and quite different from those of PNs (Rainnie et al., 2006). For instance, PV-cells in adult rats have more positive resting membrane potentials (–60 mV) than PNs and their burst-firing pattern requires high energy while other IN subtypes display regular and fast-firing patterns (Rainnie et al., 2006). The high frequency of action potentials observed in PV-positive INs and their dense excitatory innervation (Gulyás et al., 1999) makes them more sensitive to oxidative stress because of the higher metabolic requirements (Morishita et al., 2015; Steullet et al., 2017). Their higher sensitivity to oxidative stress might also make them more vulnerable to other types of stress from the environment. This is illustrated in a recent study showing changes in PV staining intensity, but not PV neuron numbers in the amygdala after early maternal separation in adult male and female rats (Gildawie et al., 2020a; Soares et al., 2020). It is interesting to note that in this particular study, maternal separation did not change PV staining intensity or cell number in the mPFC or in subfields of the hippocampus of either males or females, suggesting that effects on PV-INs are region-specific. However, ELS increased 8-oxo-dG in PV neurons, a marker of DNA oxidation in PV cells of the BLA and PL mPFC, indicating that PV neurons in these regions might be more sensitive to oxidative stress after ELS exposure (Cabungcal et al., 2013). In the mPFC, maternal separation led to a reduction in PV expression, as measured by Western blot, that appeared earlier in juvenile females than males (Holland et al., 2014).

In other regions of the corticolimbic circuit such as the hippocampus, distinct environmental exposures (i.e., either enrichment or fear conditioning) modulate the level of expression of PV in mice without necessarily modifying PV expressing cell numbers (Donato et al., 2013).

### Development of Parvalbumin Inhibitory Interneurons

The importance of IN development and functionality within the corticolimbic circuits to regulate the inhibition:excitation ratio has been well described in previous reviews (Marín, 2012; Arruda-Carvalho and Clem, 2015; Lucas and Clem, 2018) and there is little doubt that early dysfunction in INs may underlie the emergence of several neurodevelopmental disorders (Marín, 2012) in addition to adult social dysfunction (Bicks et al., 2020) and anxiety. In rodents exposed to ELS, the rhythmic firing of neurons in the amygdala (theta and gamma oscillations range) is increased (Raineki et al., 2015), while theta and beta oscillations in the mPFC and ACC are reduced (Murthy et al., 2019; Murthy and Gould, 2020). Maturation of PV-neurons coincide with the early postnatal period when ELS has profound effects on the development of the corticolimbic circuitry. PV-positive neurons begin differentiating around PND10-14 in the mouse BLA (Dávila et al., 2008) and they reach a mature phenotype by PND30 (Berdel and Moryś, 2000), at the peak of inhibitory function in the mouse amygdala (Arruda-Carvalho et al., 2017). Exposure to ELS in the form of limited bedding (LB) conditions led to a transient increase in PV-cell density in the BLA of PND21 mice that was not observed in the mPFC (Manzano Nieves et al., 2020). By adulthood, the effect of LB on PV-cell density in the BLA was absent. This is consistent with another study in juvenile rats (PND28) under LB conditions that failed to document a significant effect of ELS on PV-positive cell density in the BLA in either males or females (Guadagno et al., 2020). The type of ELS paradigm might cause different region-specific changes in PV-INs in diverse periods of life. For instance, maternal separation in rats increased PV-cell density in the BLA only in adolescent males, but not females and this effect was not seen in the PL or IL regions of the mPFC (Gildawie et al., 2020b). Prenatal insults, such as maternal infection in late gestation has also been reported to induce a large increase in mPFC PV-positive neuron density in the offspring between PND14-28 (Boksa et al., 2016), a period that coincides with the expansion of threshold levels of inhibition important to trigger critical periods of cortical plasticity (Hensch, 2005). While the onset of critical periods is typically associated with the appearance of PV-positive neurons in several cortical areas (visual, auditory, mPFC), closure of these critical periods is more closely associated with maturation of PV neurons and their stable integration in functional circuit networks. An important component of the regulation of PV maturation and PV expression is the formation of PNNs that surround perisomatic synapses and proximal dendrites of particular subsets of neurons. The role of PNNs in stabilizing synaptic inputs preferentially on PV-positive IN population (Freedom et al., 2014; Carceller et al., 2020) and in allowing for the binding of Otx2, a factor that is necessary for PV-cell maturation in the visual cortex (Bernard and Prochiantz, 2016) makes these molecular scaffolds important potential targets to ELS-induced cellular and network modifications (Kwok et al., 2011; Reichelt et al., 2019).

### Development of Perineuronal Nets (PNNs) in Corticolimbic Regions

Perineuronal nets (PNNs) are lattice-like structures consisting of large molecular aggregates composed primarily of chondroitin sulfate proteoglycans (neurocan, brevican, aggrecan, and versican) and extracellular matrix components such as collagen, laminin and fibronectin as well as additional molecules (Tsien, 2013; Van’t Spijker and Kwok, 2017). Components of PNNs are thought to be contributed by both neurons and glial cells (Carulli et al., 2006; Tanti et al., 2020) although their development is not yet fully understood. Evidence in animals suggest that PNNs may serve very different functions in specific brain regions, for example, loss of PNNs in the adult mouse visual cortex enhances LTP formation (de Vivo et al., 2013) whereas similar manipulations in the adult mouse LA reduces LTP (Gogolla et al., 2009). Furthermore, PNNs have been considered like “punch cards, in which the position and size of the holes (in the net) preserve the long-term location and strength of synapses” (Tsien, 2013). After a synapse is surrounded by the PNN, little synaptic reorganization occurs (McRae et al., 2007) and the synapse is stabilized into a mature state with limited AMPAR movement (Van’t Spijker and Kwok, 2017; Bosiacki et al., 2019). The intensity of PNN staining is thought to be correlated with the maturation level of the PNN itself; as the holes tighten their grip on the perforating synapses throughout maturation and staining intensity increases (Sigal et al., 2019). PNNs protect fast-spiking PV-INs that are susceptible to oxidative stress (Cabungcal et al., 2013; Brenhouse and Schwarz, 2016) and provide a local buffer for cations that are close to the synapse. Although PNNs preferentially surround PV-INs, other neurons, including glutamatergic neurons also harbor PNNs, but likely to a smaller extent. Parvalbumin neurons surrounded by PNNs exhibit larger somata and higher level of PV expression compared to those not ensheated with PNNs (Enwright et al., 2016). High PV expression increases the calcium buffering potential of PV-positive cells and affects GABA release from these cells. In addition to their multiple cellular roles at the level of the synapse and in particular during developmental periods, recent data have suggested that the role of PNNs might extend into adulthood to modulate some of the cellular consequences of environmental changes. Indeed, maternal experience as a surrogate mother was found to increase the density of PNNs in the mouse primary somatosensory cortex and this increase was hemispheric dependent as a function of the specific cortical region considered (Lau et al., 2020). In this study, laterality of PNN expression was observed in naïve adult mice and disappeared once the mice became surrogate mothers, suggesting that the increased demand for tasks involving tactile stimulation in surrogate mothers might have triggered activity-dependent changes in PNNs that are potentially hemispheric differentiated. More generally, it is also tempting to entertain the idea that lateralization of PNN expression in the adult brain could contribute to functional hemispheric specialization by differentially regulating INs and even principal neurons.

It is not surprising that PNNs development and maturation are activity dependent (McRae et al., 2007), coinciding directly with the closure of critical periods of plasticity in several brain regions. In the visual cortex of the mouse, closure of critical plasticity occurs at the end of the first postnatal month (Hensch, 2003) and degradation of PNNs in the adult visual cortex results in atypical or “juvenile” ocular dominance plasticity (Pizzorusso et al., 2002) and enhanced LTP formation (de Vivo et al., 2013). Within the mouse BLA, PNNs emerge on PND16 and reach mature levels by PND28 (Gogolla et al., 2009) and in rats, PNN density gradually increased between juvenile and adult rats with no obvious sex-related differences in naïve rats (Gildawie et al., 2020a). In juveniles, the percentage of PNNs on total inhibitory GAD67-positive cells in the BLA approximated 40% (Guadagno et al., 2020). By adulthood, the majority of PNNs encapsulate PV-INs as well as a fraction of excitatory cells (Alpár et al., 2006; Morikawa et al., 2017). Interestingly, the appearance of PNNs in the mouse BLA coincides with a developmental switch in fear learning, such that conditioned fear memories are subject to context-dependent renewal following extinction training (Gogolla et al., 2009). The experimental degradation of PNNs on all BLA cell types in adulthood decreases LTP formation at LA inputs, reactivates critical period of plasticity and re-enables the permanent erasure of fear memories, a process normally only observed in neonatal mice. These results suggest that in the adult BLA, PNNs actively protect fear memories from being extinguished by facilitating synaptic strengthening (Gogolla et al., 2009). Degradation of PNNs in the hippocampus or mPFC also impairs the formation of conditioned fear memories in rats (Hylin et al., 2013).

In the mPFC, increased inhibition during adolescence is thought to contribute to a sensitive period for cortical plasticity that is associated with reaching adult-like levels of PV-positive cell density in this region (Baker et al., 2017). In contrast, the maturation of PNNs drastically increases between the juvenile period and adolescence in both IL and PL subregions of the mPFC. As a result, the number of PV-positive cells expressing PNNs reaches adult-like levels already by adolescence in the rat (Baker et al., 2017). The precise development of PNNs in the mPFC might follow a slightly different trajectory between male and female rats. In males, puberty did not affect the density of PNNs, but in females, the onset of puberty led to an abrupt reduction in the number of PNNs that persisted through mid-adolescence, before showing an increase toward adult levels at PND60 (Drzewiecki et al., 2020). This sex-dependent protracted PNN development in females in adolescence was observed in both IL and PL mPFC and might indicate a longer period of cortical plasticity in the mPFC. The rise in pubertal estrogens appears necessary for an increased inhibition within the ACC, although it is still unclear how estrogens modulate the expression of PNNs in these cortical regions (Piekarski et al., 2017).

Similar to rodents, PNNs develop in human PFC and hippocampus from as young as 2 months after birth with a protracted course of postnatal maturation stabilizing between the ages of 8–12 years old (Mauney et al., 2013; Rogers et al., 2018). In the PFC, PNNs enwrap mainly PV-cells (Rogers et al., 2018), although they are expected to also enwrap excitatory cells in CA2 of the hippocampus and deep cerebellar nucleus as they do in rodents (Van’t Spijker and Kwok, 2017; Bozzelli et al., 2018). Moreover, 31% of PV-positive cells in the human amygdala are ensheathed by PNNs (Pantazopoulos et al., 2006) leaving a large population of neurons that are covered to be characterized. Although human PNN studies are lacking, alterations in PNNs have been reported in post-mortem studies of various brain disorders. Deficits in PNNs are well documented in schizophrenia across many different brain regions, including the LA, superficial layers of the entorhinal cortex, HPC and layer III and V of the PFC (reviewed in Berretta et al., 2015). Furthermore, the intensity of labeling of both PNNs and PV + cells in the dorsolateral PFC (Enwright et al., 2016) is decreased which is thought to contribute to the dysfunction of microcircuits associated with this mental illness. In bipolar disorder, PNN reduction has been observed in entorhinal cortex layer II, but not in the PFC (Pantazopoulos et al., 2010; Mauney et al., 2013). Recently, Crapser et al. (2020) reported a specific decrease in PNNs in the subiculum of Alzheimer’s disease patients. Despite these observations, little is currently known about the mechanisms underlying such disorder-specific changes nor about their functional consequences. It can be assumed, however, that changes in PNN/PV GABAergic neurotransmission may be at the root of altered gamma oscillations, an important rhythm in the BLA, which have been documented in multiple brain disorders (McNally and McCarley, 2016; Bozzelli et al., 2018; Blanco and Conant, 2020).

### Early Life Stress Affects the Formation of PNNs

**FIGURE 3:**
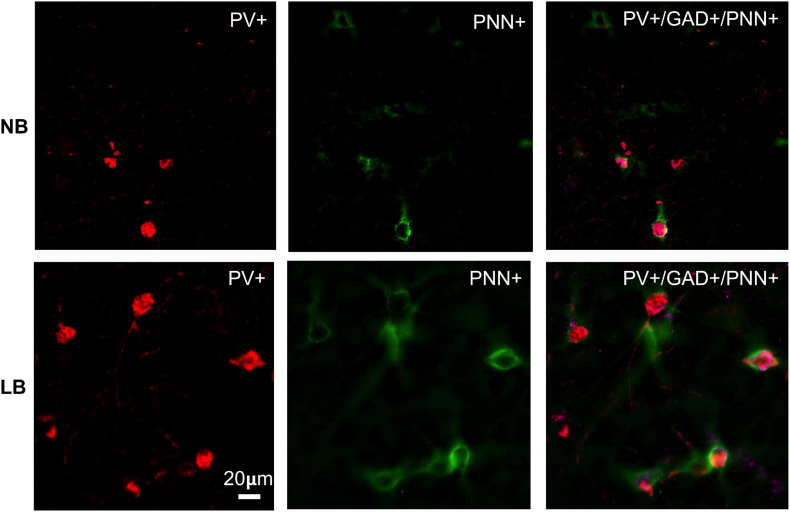
Immunohistochemical detection of parvalbumin (PV) positive neurons in the juvenile (PND28) rat BLA that are surrounded by perineuronal nets (PNN) in offspring exposed to normal bedding (NB) or limited bedding (LB) conditions during the first 10 days of postnatal life. Note the higher density of cells co-expressing PV and PNN in the BLA of LB compared to control (NB) offspring. Images were taken on an Olympus BX63 microscope at 20x magnification. Scale bar is 20 μm.

Because PNNs play an important role in regulating the activity of and synaptic inputs onto the neurons they surround, alterations in PNN structure and/or density by ELS may have strong consequences for neural functioning (Sorg et al., 2016; Reichelt et al., 2019). Exposure to ELS has been linked to aberrant PNN density and intensity in all regions included in the corticolimbic circuit (Riga et al., 2017; Page and Coutellier, 2018, 2019; Santiago et al., 2018), although the direction of effects is not always consistent between studies and brain regions. In rodents subjected to daily maternal separation (4 h/day) during the neonatal period, a reduction in PV cell density was observed in the mPFC of adolescent males, but not females (Soares et al., 2020) although PNN density was reduced in both sexes at PND40 (Gildawie et al., 2020a). The reduction in PNN density was observed mainly in the IL mPFC and not in the PL area. In contrast, the BLA of maternally separated rats exhibited increased PV and PNN density in adolescence, but only in males (Gildawie et al., 2020a). Despite the increased PV and PNN density in the BLA after maternal separation, the intensity of PV staining was reduced by ELS and correlated with an increase in 8-oxo dG staining, a marker of DNA oxidation (Soares et al., 2020). This suggests that maternal separation might affect the structure of PNNs, making them less efficient at protecting PV-neurons from oxidative stress. Indeed, prolonged periods of maternal separation paired with early weaning (MSEW) in mice altered the structural integrity of PNNs enwrapping PV-neurons in the vHipp of adult males (Murthy et al., 2019). In this region, MSEW reduced PV-cell density as well as the intensity of PV staining, but PNN density was unaltered and PNN intensity around PV-positive neurons in the subregion of the ventral dentate gyrus was actually increased, indicating that changes in PNN intensity might not always vary in parallel with changes in density. In other types of ELS, like the limited bedding conditions, PNN density was increased in the BLA of juvenile male rats and this effect was lateralized to the right amygdala exclusively (Guadagno et al., 2020; Figure 3). The increase in PNN density was not accompanied by a significant increase in PV-cell density in either sex, although the total proportion of PV-cells surrounded with PNN was increased in males (Guadagno et al., 2020), suggesting that the increase in PNN observed after the early stress of limited bedding targeted mostly PV-neurons.

**FIGURE 4:**
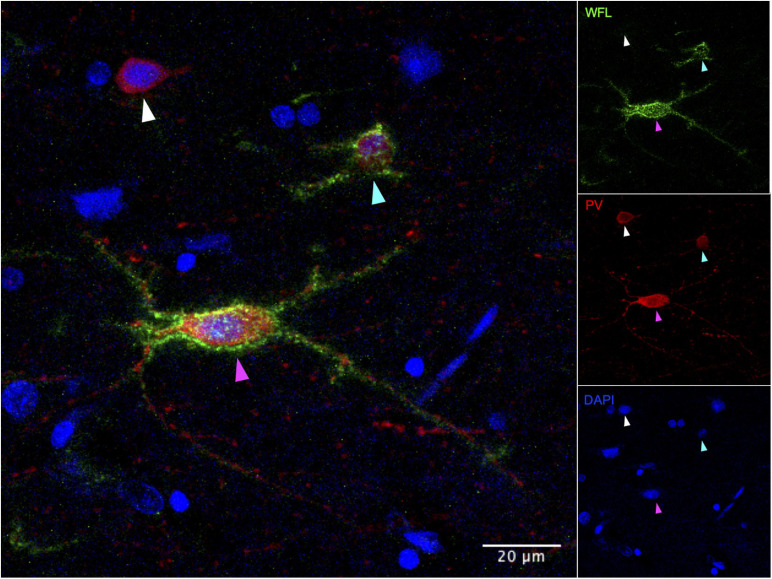
Immunohistochemical detection of parvalbumin positive neurons in post-mortem human vmPFC that are surrounded or not by perineuronal nets. WFL, Westeria Floribunda Lectin; PV, Parvalbumin; DAPI, nuclear staining. White arrow: PV+/PNN– cell, Cyan arrow: PV+/PNN+ low intensity/coverage, Magenta arrow: PV+/PNN+ high intensity/coverage. Images were taken on an Olympus FV1200 laser scanning confocal microscope at 40x magnification.

To our knowledge, a single study has examined the possible consequences of ELS on PNNs in human post-mortem tissue. This recent paper reported an increase in PNNs in layers III, IV, V, and VI of the vmPFC of adult depressed suicides with a history of severe child abuse (Tanti et al., 2020). Along with the augmented density of PNNs, the authors observed an increase in intensity of the staining and coverage of enwrapped cells (Figure 4) that was associated with child abuse. Using *in situ* hybridization, the same study found that 65% of vmPFC PV-positive cells were surrounded by PNNs in psychiatrically healthy controls (Tanti et al., 2020) which is similar to the ratio calculated in rodent studies using IHC (Van’t Spijker and Kwok, 2017). The ratio of neurons enwrapped by PNNs was disturbed in depressed suicides with a history of child abuse. This study suggests that ELS from sexual, emotional or physical abuse is associated with precocious maturation and increased recruitment of PNNs in the human vmPFC, which is consistent with the aforementioned stress acceleration hypothesis (Callaghan and Tottenham, 2016).

Together, the few studies that have examined the effects of ELS on the density and intensity of PNNs have raised important questions that will need to be addressed in the future. For instance, what are the mechanisms underlying regional differences in PV/PNN sensitivity to ELS? Are cell populations other than PV-positive inhibitory INs more likely to be enwrapped by PNNs in response to ELS? It is suggested that in normal BLA development, the increase in PNN density might be contributed to by an increase in PNN surrounding non-PV cells preferentially (Baker et al., 2017). Regardless of PNN density and intensity changes, is the molecular composition of the PNNs changing with ELS? Is there a PNN composition that is optimal for synaptic stabilization? PNNs surrounding certain cells (most likely pyramidal) appear more delicately/weakly stained in comparison to those surrounding PV-positive cells in the cortex; it will be important to determine whether the enwrapped cell type dictates PNN composition and whether ELS modifies this relationship between PNN intensity/composition and cell type.

### Summary

Complex local intra-amygdalar circuits with multiple interactions between PNs and several classes of INs as well within INs subtypes are fine-tuning the behavioral responses to aversive stimuli. In the amygdala and in particular in the BLA, 50% of the inhibitory INs are PV-cells that densely innervate excitatory neurons. Their PV expression and high firing activity is fully mature by PND30, at the peak of inhibitory activity in this structure. PV-neurons are surrounded by PNNs that protect them from oxidative stress and stabilize synaptic inputs. Interestingly, maturation of PNNs not only signals the termination of critical periods of plasticity within corticolimbic structures, but also allows for greater PV expression and GABAergic neurotransmission. It is perhaps not surprising to observe that ELS increases the expression of PNNs in the rodent BLA since PNN production is activity dependent. Increased PNN expression has also been documented after a history of child abuse in the human vmPFC. In contrast, in the rodent mPFC, ELS tends to reduce PNN expression and this might maintain PV-inhibitory neurons in a “labile” state longer than in the BLA, allowing for modifications and adaptations in neuronal input for longer time periods. Overall, multiple cellular processes participate in the organization and developmental regulation of corticolimbic activity, with plasticity in the formation of synaptic inputs terminating when the proper cellular scaffoldings are in place to insure stable and efficient inhibitory neurotransmission within local circuits. The fact that ELS significantly affects these specific cellular processes might constitute an additional target for treatment of aberrant fear memories and heightened states of anxiety in young and adult patients. In this respect, experiments showing that PNN dissolution in adult rodents could restore a developmental fear memory “phenotype” with the erasure of fear could constitute a promising mechanism to erase traumatic memories in PTSD and anxious patients. Similarly, the targeting of specific IN populations based on their phenotype and specific action on PNs might represent a useful addition to current psychopharmacological treatments.

## Conclusion and Future Directions

The dramatic and long-lasting consequences of childhood adversity and ELS have now been extensively documented in emotional, cognitive and social areas across species and sexes. The urgency to identify risk, but also resilience phenotypes after exposure to ELS has stimulated a large research effort with the overall goal to understand mechanisms, identify critical windows of susceptibility and plasticity, allowing for targeted and efficient interventions to curb the occurrence and severity of negative consequences. Functional connectivity and imaging studies from rodents to humans have consistently found region-specific and network-specific differences in the effects of ELS, in particular within the corticolimbic circuit. Accelerated development of the BLA and/or enhanced excitability in this structure have been proposed to drive the development of reciprocal BLA-PFC projections, modify cellular components of the network, and possibly reorganize critical periods of plasticity through effects on INs and PNNs. Pathways governing fear extinction are significantly altered after ELS, leading to the emergence of prolonged anxiety and other comorbid disorders. Here again, the formation of PNNs around INs is an important process that will set their connectivity and reduce their plasticity. Recovering a “labile” state of IN activity by altering specific components of the PNNs might represent an interesting future therapeutic prospect in the treatment of individuals with impaired fear extinction for instance.

The large number of studies performed on non-human primates and rodents have informed many of these processes, but also raised important questions that will need to be answered. For instance, how does ELS modify critical periods of plasticity (Reh et al., 2020) in the corticolimbic circuit in a risk and resilient phenotype? Are there cellular and molecular markers of vulnerability that reflect the variability in the outcomes of ELS? What are the molecular mechanisms through which ELS are affecting PNNs and local network activity in a region-specific manner? How does sex bias in preclinical experimental models reflect the observed sex-dependent cognitive and emotional consequences in humans? Future experiments should focus on elucidating potential mechanisms to explain sex differences in outcomes, investigating the role of early organizational effects of gonadal steroids and/or sex chromosomes on morphology, inhibitory tone and functional connectivity, identifying the primary IN subtypes that drive ELS effects and identifying ways to specifically target these, maybe through indirect effects on PNN integrity. Hopefully, answers to these questions will not only further our understanding of molecular and cellular processes underlying fear behavior, but also inform clinical and psychosocial teams to find the best time and modality of intervention to alleviate some of the dramatic consequences of early childhood adversity and trauma. We collectively owe this to our children.

## Author Contributions

AG and C-DW wrote the first draft of the manuscript, and edited and reviewed the final version. CB and NM wrote sections of the manuscript, and edited and reviewed the final version. All authors contributed to the article and approved the submitted version.

## Conflict of Interest

The authors declare that the research was conducted in the absence of any commercial or financial relationships that could be construed as a potential conflict of interest.

## Publisher’s Note

All claims expressed in this article are solely those of the authors and do not necessarily represent those of their affiliated organizations, or those of the publisher, the editors and the reviewers. Any product that may be evaluated in this article, or claim that may be made by its manufacturer, is not guaranteed or endorsed by the publisher.
